# The Impacts of Read Length and Transcriptome Complexity for *De Novo* Assembly: A Simulation Study

**DOI:** 10.1371/journal.pone.0094825

**Published:** 2014-04-15

**Authors:** Zheng Chang, Zhenjia Wang, Guojun Li

**Affiliations:** School of Mathematics, Shandong University, Jinan, Shandong, China; The Rockefeller University, United States of America

## Abstract

Transcriptome assembly using RNA-seq data - particularly in non-model organisms has been dramatically improved, but only recently have the pre-assembly procedures, such as sequencing depth and error correction, been studied. Increasing read length is viewed as a crucial condition to further improve transcriptome assembly, but it is unknown whether the read length really matters. In addition, though many assembly tools are available now, it is unclear whether the existing assemblers perform well enough for all data with different transcriptome complexities. In this paper, we studied these two open problems using two high-performing assemblers, Velvet/Oases and Trinity, on several simulated datasets of human, mouse and S.cerevisiae. The results suggest that (1) the read length of paired reads does not matter once it exceeds a certain threshold, and interestingly, the threshold is distinct in different organisms; (2) the quality of *de novo* assembly decreases sharply with the increase of transcriptome complexity, all existing *de novo* assemblers tend to corrupt whenever the genes contain a large number of alternative splicing events.

## Introduction

RNA-seq provides a powerful tool to reveal the complex landscape and dynamics of transcriptomes at an unprecedented level of sensitivity and accuracy [1]–[4]. Compared with microarray data and EST sequencing, RNA-seq has a lot of advantages, such as single nucleotide resolution, higher dynamics range to quantify gene expression level, and ability to distinguish rare transcripts and alternative splicing transcripts [2], [5], [6]. However, the sequence reads obtained from RNA sequencing tend to be very short and error-prone [7], hence posting tremendous computational challenges to analyze transcriptome from the RNA-seq reads.

To obtain an accurate and completed transcriptome is a prerequisite to most of, if not all, downstream transcriptomic analyses, such as differential expression, functional genomics, SNP discovery, etc. In the last few years, a number of transcriptome assemblers have been developed, which can be classified into two general categories: reference-based approaches [8], [9] and *de novo* assembly approaches [10]–[12]. Though reference-based approaches perform better [11]–[13], *de novo* assembly provides an important solution when the reference genome is not available, or incomplete, or substantially varied as in cancer cells.

Though the vast majority of computational research has been focused on assembly methods, only limited knowledge regarding the impact of sequencing data on *de novo* assembly has been known. Recently, Warren Francis *et al.* [14] studied the effect of sequencing depth and gave an optimal sequencing depth for *do novo* transcriptome assembly. Another research paper [15] studied how error correction impacts assembly accuracy. Increasing read length of Illumina RNA-seq data is currently viewed as a crucial condition to further improve the quality of *de novo* transcriptome assembly, but it is unknown whether the read length really matters. How does the read length affect *de novo* assembly? Mark Chaisson *et al.* [16] provided a very surprising answer to this problem in the context of the genome assembly with mate-paired reads, which says that the read length does not matter once the read length exceeds a certain threshold. But for transcriptome assembly, as far as we know, nobody has ever studied whether the read length matters, or whether such a threshold exists.

*De novo* transcriptome assembly has been dramatically improved over the last few years, but it is unknown whether the existing assembly methods have been good enough for all kinds of data. Due to alternative splicing, transcriptome assembly requires a graph structure, opposite to a linear sequence like in genome assembly, to provide a complete representation of multiple spliced isoforms per locus and then all the isoforms can be derived from this graph. More alternative splicing events would dramatically increase the complexity of the graph, resulting in more challenges in the step of searching isoforms from the graph. Intuitively, we suspect that the transcriptome complexity should have a significant impact on the quality of *de novo* assembly. However, until now, no public research has ever been particularly interested in this problem.

In this paper, we focused on the two open questions mentioned above and studied the impacts of read length and transcriptome complexity on the quality of *de novo* assembly using simulated Illumina RNA-seq datasets of human, mouse and S.cerevisiae. The results strongly demonstrate that (1) the read length of paired-read has a large effect on assembly quality only when it is under a certain threshold which is distinct in different organisms, and once exceeding such a threshold, the read length does not matter at all; (2) the quality of *de novo* transcriptome assembly decreases sharply with the increase of transcriptome complexity, all existing *de novo* assemblers tend to corrupt whenever the genes contain a large number of alternative splicing events. Considering the significant importance of *de novo* assembly, we believe that our findings would be instructive and meaningful for further transcriptomic study.

## Results

To exploit the impacts of read length and transcriptome complexity on the quality of *de novo* assembly, we compared the *de novo* assemblies on simulated datasets with different read lengths and with different transcriptome complexities, respectively. Two high-performing assemblers, Velevt/Oases [11], [17] and Trinity [12], were used to guarantee that our results are by no means fortuitous. We used simulated data, instead of real data, due to the following two considerations: first, assessing the quality of *de novo* assembly requires the knowledge of the true transcriptome, which is not possible for the real data; second, real datasets with different lengths cannot be obtained by simply cutting long reads short because the error rate is not consistent across the whole sequencing read. Short reads cut from the end of the sequencing reads usually have a higher sequencing error rate, which would affect the interpretation of the experiment result.

### Does read length matter?

The read length of illumine RNA-seq data has become longer and longer with the development of sequencing technology. One open question to be studied is whether the read length really matters. Though longer reads are thought to be able to improve the assembly quality, there is no evidence showing that the quality of transcriptome assembly improves as the read length goes up.

**Figure 1:**
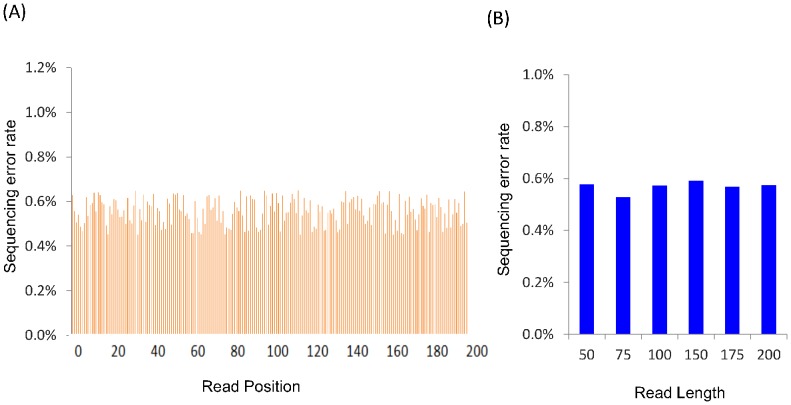
Distribution of sequencing errors. (A) shows the sequencing errors occur randomly across one read and B) shows the error rate is almost the same among six human datasets with different read lengths.

We first generated six simulated RNA-seq datasets of human by a simulation engine called BEERS [18]. Each dataset consists of ∼50 million paired-reads, with an insert size of 200 bp but with different read lengths of 50 bp, 75 bp, 100 bp, 150 bp, 175 bp and 200 bp (see Methods and Data S1). The sequencing error rate is randomly distributed across one read (Figure 1A), and is almost the same among different datasets (Figure 1B). The sequencing depth is adequate for *de novo* assembly according to the study of Warren Francis *et al* [14]. All of these considerations will minimize, if not avoid, the effects of sequencing errors and sequencing depth.

**Figure 2:**
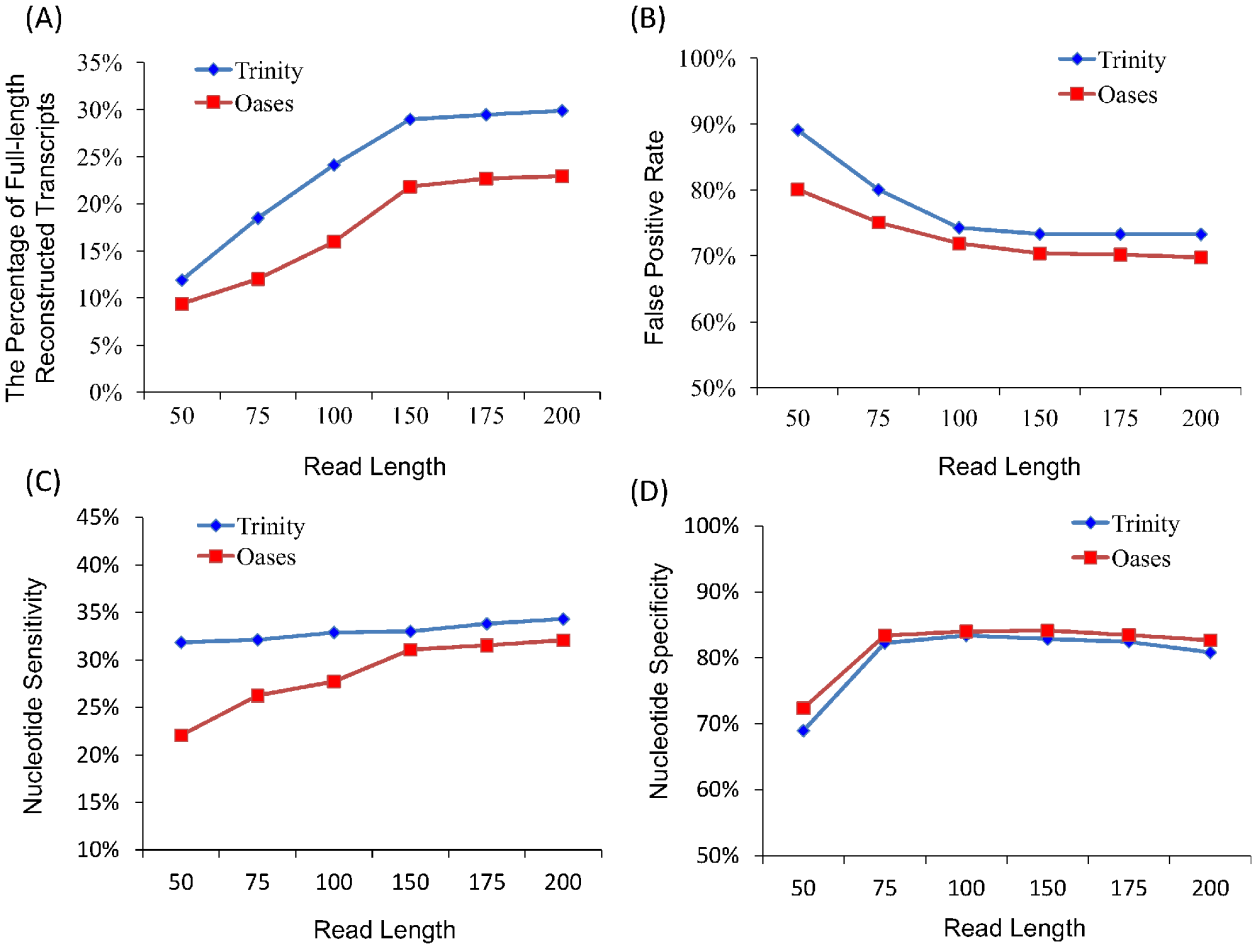
Assessing the quality of *de novo* assemblies on human datasets with different lengths using four different measures: (A) the percentage of full-length reconstructed reference transcripts, (B) false positive rate, (C) nucleotide sensitivity, and (D) nucleotide specificity.

The quality of assembly is evaluated by the following four metrics: the percentage of full-length reconstructed reference transcripts, false positive rate, nucleotide sensitivity, and nucleotide specificity (see Methods and Data S2). The results of Trinity and Oases are shown in Figure 2. Basically, Trinity has higher percentage of full-length reconstructed transcripts (Figure 2A) and higher nucleotide sensitivity (Figure 2C) than Oases, but with higher false positive rate (Figure 2B) and a little lower nucleotide specificity (Figure 2D). In spite of the different performances, both assemblers consistently suggest that the quality of transcriptome assembly improves significantly with the read length increasing from 50 bp to 150 bp, and does not improve any more after that, in the sense of the following four facts (Table S1): (i) the percentage of full-length reconstructed reference transcripts increases no more than 1%; (ii) the false positive rate does not decrease; (iii) the nucleotide sensitivity only improves by about 1%; and (iv) the nucleotide specificity even exhibits a little decrease (about ∼0.8%).

Based on these observations, we can conclude that the read length does matter only when it is under a certain threshold, which is ∼150 bp for the human datasets. Once the read length exceeds such a threshold, increasing the read length does not contribute to the assembly quality any more (less than 1% improvement in all measures).

**Figure 3:**
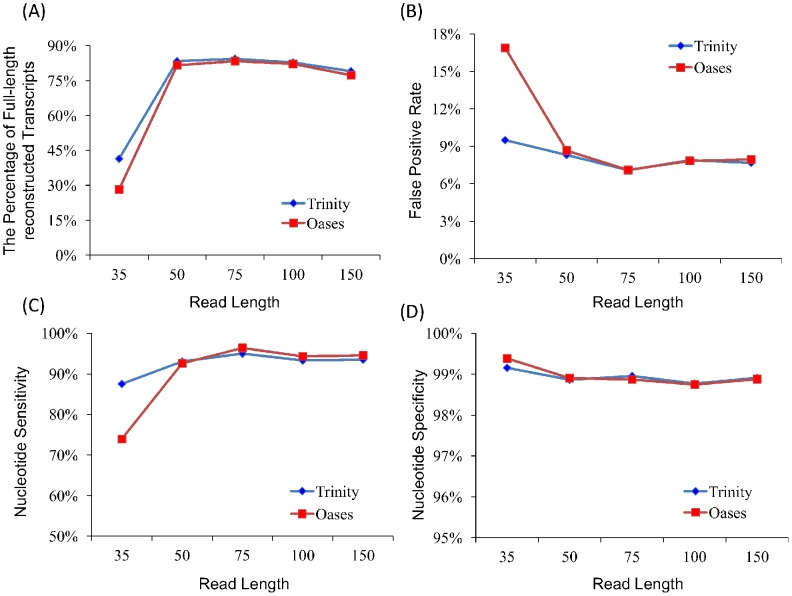
Four assessment metrics of assemblies on S.cerevisiae datasets with different lengths.

We also simulated five S.cerevisiae datasets to check whether the read length could have different impacts on distinct organisms. Each dataset contains 40∼50 million paired-reads with an insert size of 200 bp but with different read lengths of 35 bp, 50 bp, 75 bp, 100 bp and 150 bp (see Methods). Trinity and Oases were run on these datasets and their results were evaluated by the same measures as used on human. It is obvious that there also exists a threshold (∼75 bp) in S.cerevisiae after which the increase of read length does not matter at all (Figure 3 and Table S2). An interesting observation is that the threshold in S.cerevisiae is much less than that in human, indicating that there indeed exist different thresholds in different organisms. The possible reason of smaller threshold in S.cerevisiae is that its transcriptome is too simple to have ambiguities resulted from alternative splicing that need to be resolved by long reads.

**Figure 4:**
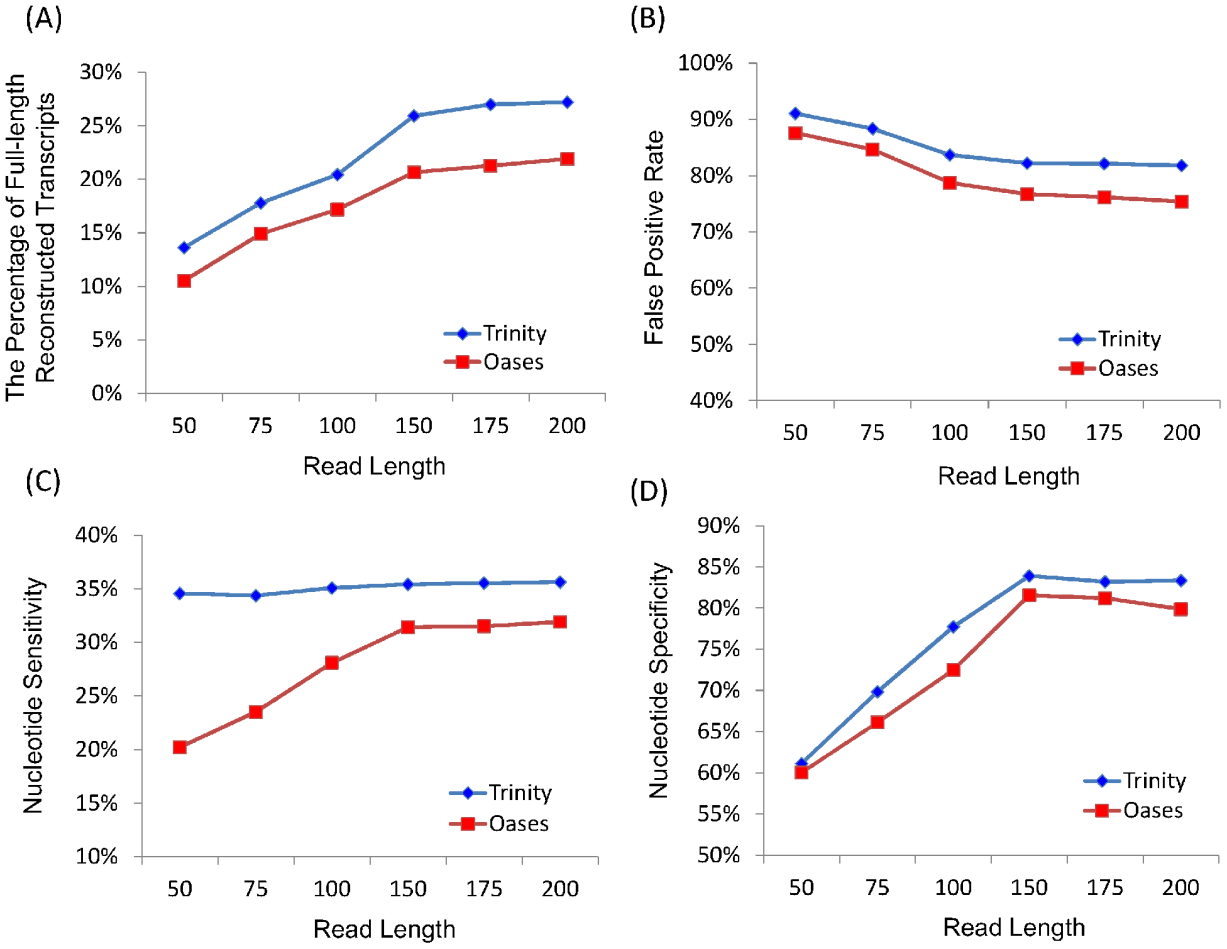
Assessing the quality of *de novo* assemblies on mouse datasets with different lengths.

Is the threshold the same in two similar species? To answer this question, we simulated six mouse datasets with an insert length 200 bp (see Method and Data S3). The range of read length is the same as human data, from 50 bp to 200 bp. As expected, both Trinity and Oases indicate there exists a threshold in mouse (Figure 4 and Table S3). Interestingly, the threshold is also 150 bp, the same as the threshold in human. This is because the complexities of transcriptomes of human and mouse are similar to each other.

Although the read length of RNA-seq data is becoming longer and longer, our study demonstrates that long reads may not always help to improve the quality of *de novo* transcriptome assembly. Therefore, one should not blindly pursue longer reads even at the cost of higher sequencing error rate. Actually, we also simulated different sequencing error rates by a perl script for the S.cerevisiae dataset with read length of 75 bp (Data S2). The result of Trinity indicate that the sequencing error rate could significantly affect assembly accuracy (Table 1). For more details about the relationship between assembly and sequencing error, please refer to the study of MacManes *et al.* [15].

**Table 1:** Comparison of *de novo* assemblies on S.cerevisiae datasets with different sequencing error rates (the read length is 75 bp).

Error Rate	aFull-length Percentage	False Positive Rate	Nucleotide Sensitivity	Nucleotide Specificity
error-free	84.4%	7.1%	95%	99.4%
0.5%	84.3%	7.1%	94.8%	98.9%
1%	82.8%	7.8%	94.3%	98.5%
2%	79.1%	10.3%	93.5%	95.4%

a Full-length Percentage: the percentage of full-length reconstructed reference transcripts.

### The impact of transcriptome complexity on *de novo* assembly

Alternative splicing is a normal and nearly universal phenomenon in mammals like human [19], [20]. Intuitively, more alternative splicing events would dramatically increase the challenges of constructing transcripts. By now, nobody has ever studied the relationship between transcriptome complexity and the quality of *de novo* assembly.

**Figure 5:**
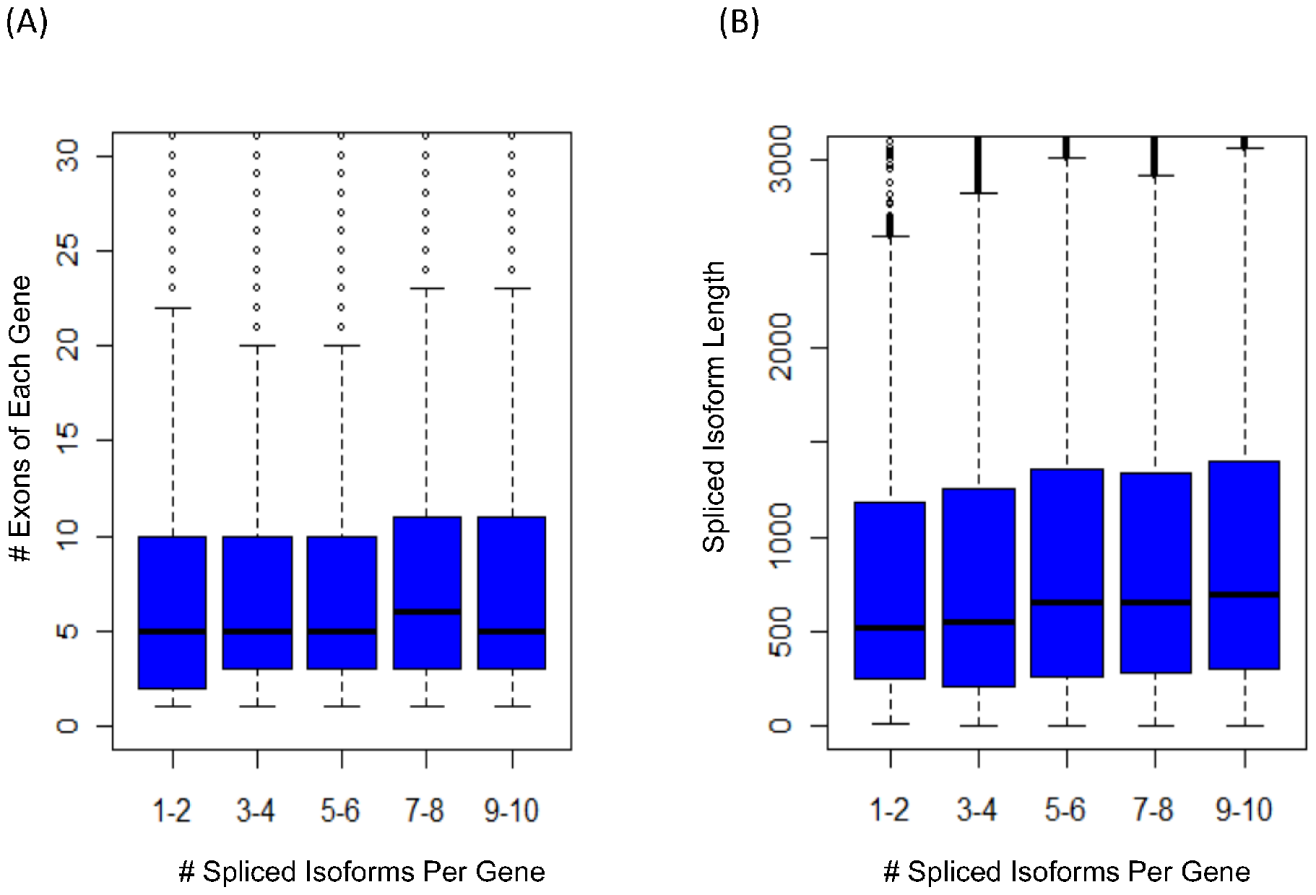
Analyses of five human datasets with different numbers of spliced isoforms. (A) Boxplot of exon number of each gene. (B) Boxplot of spliced isoform length.

To address this problem, we measured transcriptome complexity as the average number of spliced isoforms of each gene and simulated five human datasets with different numbers of spliced isoforms. The read length (100 bp) and insert size (200 bp) of paired reads are the same among different datasets. Each of the five datasets contains the same number of genes, but with various spliced isoform numbers ranging from 1∼2 to 9∼10 isoforms per gene (see Methods and Data S4). Neither the exon number of each gene nor the isoform length has significant difference among different simulated datasets (Figure 5). The number of paired reads (ranging from 9 to 57 millions) for each dataset is proportional to the number of transcripts to guarantee a comparable sequencing depth (see Table 2).

**Table 2:** Statistics of five human datasets with different numbers of spliced isoforms.

DatasetIDs	# Isoforms/Gene	# Genes	# Transcripts	Total Reads	# Reads/Transcript
1	1∼2	4000	6000	9 M	1500
2	3∼4	4000	14000	21 M	1500
3	5∼6	4000	22000	33 M	1500
4	7∼8	4000	30000	45 M	1500
5	9∼10	4000	38000	57 M	1500

**Figure 6:**
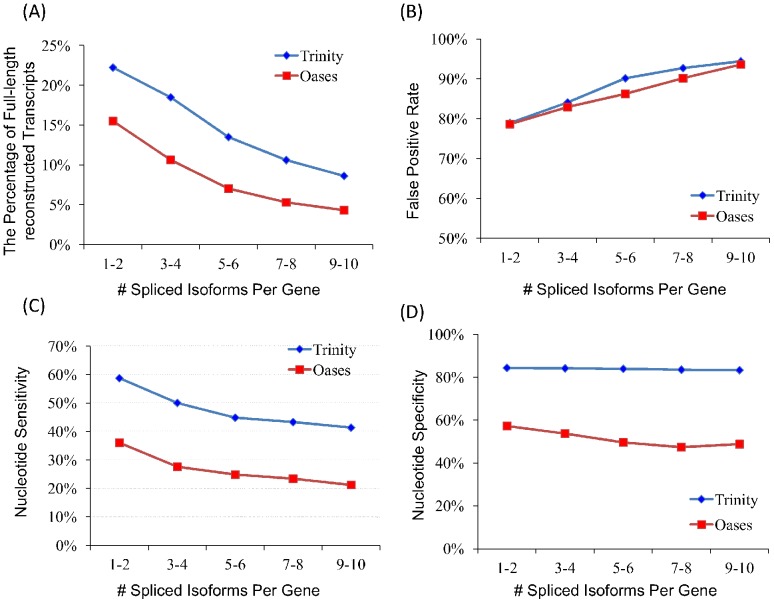
Comparison of *de novo* assemblies on five human datasets with different numbers of spliced isoforms.

The evaluations of Trinity and Oases on these datasets using four different metrics are illustrated in Figure 6. Though Trinity performs better than Oases, both assemblers consistently demonstrate that the quality of *de novo* transcriptome assembly decreases sharply with the increase of transcriptome complexity, particularly in the sense of two most important measures: the percentage of full-length reconstructed reference transcripts (Figure 6A) and false positive rate (Figure 6B). In fact, when the average spliced isoform number of each gene in the transcriptome is up to 9∼10, only less than 5% of reference transcripts could be full-length reconstructed, and the false positive rate is even higher than 93% (Table S4), which implies a meaningless assembly. Further assessing the different assemblies in the nucleotide level, we can see that the nucleotide sensitivity also decreases with the increasing number of spliced isoforms (Figure 6C), while the nucleotide specificity is relatively stable (Figure 6D).

Though *de novo* transcriptome assembly has been dramatically improved, all existing *de novo* assemblers tend to corrupt whenever the genes contain a large number of alternative splicing events. This observation shows that the assembly method still need to be further improved by more sophisticated computational technologies, which could particularly address the genes with many alternative splicing events.

## Methods

### Preparing datasets

We used the following simulated RNA-seq datasets to exploit the impacts of read length and transcriptome complexity on the quality of *de novo* assembly (All datasets can be regenerated by the instructions in Data S1, S2, S3 and S4).

1. Six datasets of human with different lengths of 50 bp, 75 bp, 100 bp, 150 bp, 175 bp, 200 bp. Each of these datasets contains ∼50 million paired reads with an insert size of 200 bp generated by a simulation engine called BEERS [18]. BEERS first randomly chooses 10,000 genes from 11 different published annotation efforts (AceView, Ensembl, Geneid, Genscan, NSCAN, OtherRefSeq, RefSeq, SGP, Transcriptome, UCSC, Vega), and then introduces sequencing errors and alternative splice isoforms (see Data S1).
2. Five datasets for S.cerevisiae with lengths of 35 bp, 50 bp, 75 bp, 100 bp, 150 bp. Each dataset consists of 40∼50 million paired reads with an insert size of 200 bp. Since BEER can only simulate reads for human or mouse, we generated these datasets by ourselves as follows. First, 6,713 reference transcripts of S.cerevisiae are downloaded from Ensemble (http://www.ensembl.org/info/data/ftp/index.html), and then a random expression level is assigned to each transcript. Finally, paired reads are generated from these transcripts based on their expression levels (see Data S2).
3. Six mouse datasets with different lengths of 50 bp, 75 bp, 100 bp, 150 bp, 175 bp, 200 bp. Each datasets has ∼50 million paired reads generated by BEERS, with an insert size of 200 bp. (see Data S3).
4. Five human datasets of human with different spliced isoforms. The read length (100 bp) and insert length (200 bp) of paired reads are the same among all datasets, but spliced isoforms ranging from 1∼2 isoforms per gene to 9∼10 isoforms per gene (see Data S4). These datasets are generated from the same number (4000) of genes with different numbers of transcripts ranging from 6000 to 38000 (Table 2).

### Mapping to reference

All assembled transcripts were mapped to the reference using BLAT [21] with 95% sequence identity. If one assembled transcript fully covered one reference transcript with 1% indels, we say this reference transcript is full-length reconstructed. Considering the alternative splicing, a stringent indel cutoff is used mainly to avoid the potential problem of over-estimating consistencies between predicted transcripts and the reference transcripts.

### Assessing assembly quality

Although the standards for systematically assessing the quality of transcriptome assemblies have not been established, several criteria are proposed in recent studies [11], [12], [22]. In this paper, we evaluate different assemblies using the following four metrics: (i) the percentage of full-length reconstructed reference transcripts; (ii) false positive rate, which is the ratio between the false positive predictions and the total number of assemblies, where a false positive prediction means a predicted transcript that less than 50% of its sequence can be aligned to some reference transcript; (iii) nucleotide sensitivity, which is calculated by the number of correct bases divided by the number of reference transcriptomic bases [11]; (iv) nucleotide specificity, which is the ratio between the number of correct bases and the number of predicted transcriptomic bases.

### *De novo* assembly

RNA-seq reads were *de novo* assembled using Trinity (version r2013-08-14) [12] and Velvet (version 1.2.01) + Oases (version 0.2.02) [11], [17]. The command line parameters used for Trinity is “--CPU 6 --bflyHeapSpaceMax 10G --bflyGCThreads 4” for all datasets, for Velvet is “-read_trkg yes -ins_length 200” and for Oases is: “ -min_trans_lgth 200”. The *k*-mer length is an important parameter, which is 25 in Trinity, while variable in Oases. Though Oases is a multiple-*k* assembler which can use a range of *k*-mer assemblies, we just treat it as a single-*k* assembler and take the optimal value on each dataset in our study. All the assemblies were performed on a server with 512GB of RAM. After assembly, only transcripts with no less than 200 bases were used for downstream analysis.

## Discussion

In the past few years, significant efforts have been spent on assembly method, but only limit knowledge regarding the impact of sequencing data on *de novo* assembly is known. Particularly, increasing read length is currently viewed as a crucial condition for transcriptome assembly, but it is unknown whether the read length matters. In this study, different simulated datasets of human, mouse and S.cerevisiae were generated to address this issue. The experimental results consistently suggest that the read length of paired reads does not matter once the read length exceeds a certain threshold, and interestingly, this threshold is the same in similar species and different in distinct organisms.

Intuitively, more alternative splicing events may have negative effect for assembly quality, however, as of today, no public research has ever been particularly interested in this problem. We quantitatively studied the relationship between transcriptome complexity, measured as the average number of spliced isoforms of each gene, and the quality of *de novo* assembly. The test results on five human datasets indicate that the quality of transcriptome assembly, at least based on the existing *de novo* assemblers, decreases sharply with the increase of transcriptome complexity. Therefore, the assembly method has to be further improved in the future.

An important and counterintuitive implication of our first conclusion is that one may not always blindly pursue longer reads even at a cost of higher sequencing error rate. The second conclusion reminds us to pay attention to those genes with many alternative splicing events when designing new assembly method. Based on the significant importance of *de novo* assembly, we believe that our findings would be instructive and meaningful for further transcriptomic study.

## Supporting Information

Table S1 **Comparison of** ***de novo*** **assemblies on six human datasets with different lengths.**
(DOCX)

Table S2 **Comparison of** ***de novo*** **assemblies on five S.cerevisiae datasets with different lengths.**
(DOCX)

Table S3 **Comparison of** ***de novo*** **assemblies on six mouse datasets with different lengths.**
(DOCX)

Table S4 **Comparison of** ***de novo*** **assemblies on five human datasets with different numbers of spliced isoforms.**
(DOCX)

Data S1 **Information of generating six human datasets with different lengths.**
(ZIP)

Data S2 **Information of generating five S.cerevisiae datasets with different lengths.**
(ZIP)

Data S3 **Information of generating six mouse datasets with different lengths.**
(ZIP)

Data S4 **Information of generating five human datasets with different numbers of spliced isoforms.**
(ZIP)
