# Supplementary material for: The Impacts of Read Length and Transcriptome Complexity for De Novo Assembly: A Simulation Study
Source: PLoS One. 2014 Apr 15;9(4):e94825. doi: 10.1371/journal.pone.0094825 (PMC3988101; doi:10.1371/journal.pone.0094825)
Supplement: Table S1 — Comparison of de novo assemblies on six human datasets with different lengths. (DOCX) [file pone.0094825.s001.docx]

**Table S1.** Comparison of de novo assemblies on six human datasets with different lengths using four different measures, including the percentage of full-length reconstructed reference transcripts, false positive rate, nucleotide sensitivity, and nucleotide specificity.

| Read Length | *Do novo* Assembler | Full-length Percentage | False Positive Rate | Nucleotide Sensitivity | Nucleotide Specificity |
| --- | --- | --- | --- | --- | --- |
| 50 | Trinity | 11.9% | 89.1% | 31.9% | 69.0% |
|  | Oases | 9.4% | 80.1% | 22.1% | 72.4% |
| 75 | Trinity | 18.5% | 80.1% | 32.1% | 82.3% |
|  | Oases | 12.0% | 75.1% | 26.3% | 83.4% |
| 100 | Trinity | 24.1% | 74.3% | 32.9% | 83.4% |
|  | Oases | 16.0% | 71.9% | 27.8% | 84.0% |
| 150 | Trinity | 29.0% | 73.3% | 33.4% | 82.9% |
|  | Oases | 21.8% | 70.4% | 31.1% | 84.2% |
| 175 | Trinity | 29.5% | 73.3% | 33.8% | 82.4% |
|  | Oases | 22.7% | 70.2% | 31.6% | 83.5% |
| 200 | Trinity | 29.9% | 73.3% | 34.3% | 80.8% |
|  | Oases | 22.9% | 69.8% | 32.1% | 82.7% |
