# Supplementary material for: The Impacts of Read Length and Transcriptome Complexity for De Novo Assembly: A Simulation Study
Source: PLoS One. 2014 Apr 15;9(4):e94825. doi: 10.1371/journal.pone.0094825 (PMC3988101; doi:10.1371/journal.pone.0094825)
Supplement: Table S2 — Comparison of de novo assemblies on five S.cerevisiae datasets with different lengths. (DOCX) [file pone.0094825.s002.docx]

**Table S2.** Comparison of de novo assemblies on five S.cerevisiae datasets with different lengths using four different measures, including the percentage of full-length reconstructed reference transcripts, false positive rate, nucleotide sensitivity, and nucleotide specificity.

| Read Length | *Do novo*  Assembler | Full-length Percentage | False Positive Rate | Nucleotide Sensitivity | Nucleotide Specificity |
| --- | --- | --- | --- | --- | --- |
| 35 | Trinity | 41.4% | 9.5% | 87.5% | 99.2% |
|  | Oases | 28.3% | 16.9% | 74.0% | 99.4% |
| 50 | Trinity | 83.4% | 8.3% | 93.1% | 98.9% |
|  | Oases | 81.6% | 8.7% | 92.6% | 98.9% |
| 75 | Trinity | 84.4% | 7.1% | 95.0% | 99.0% |
|  | Oases | 83.4% | 7.7% | 96.4% | 98.9% |
| 100 | Trinity | 82.8% | 7.9% | 93.3% | 98.8% |
|  | Oases | 82.2% | 7.8% | 94.3% | 98.7% |
| 150 | Trinity | 80.1% | 7.7% | 93.5% | 98.9% |
|  | Oases | 79.3% | 8.0% | 94.6% | 98.9% |
