# Supplementary material for: The Impacts of Read Length and Transcriptome Complexity for De Novo Assembly: A Simulation Study
Source: PLoS One. 2014 Apr 15;9(4):e94825. doi: 10.1371/journal.pone.0094825 (PMC3988101; doi:10.1371/journal.pone.0094825)
Supplement: Table S3 — Comparison of de novo assemblies on six mouse datasets with different lengths. (DOCX) [file pone.0094825.s003.docx]

**Table S3.** Comparison of de novo assemblies on six mouse datasets with different lengths using four different measures, including the percentage of full-length reconstructed reference transcripts, false positive rate, nucleotide sensitivity, and nucleotide specificity.

| Read Length | *Do novo* Assembler | Full-length Percentage | False Positive Rate | Nucleotide Sensitivity | Nucleotide Specificity |
| --- | --- | --- | --- | --- | --- |
| 50 | Trinity | 13.6% | 91.0% | 34.6% | 61.6% |
|  | Oases | 10.5% | 87.6% | 10.2% | 60.0% |
| 75 | Trinity | 17.8% | 88.3% | 34.4% | 69.8% |
|  | Oases | 14.9% | 84.6% | 23.5% | 66.1% |
| 100 | Trinity | 20.4% | 83.7% | 35.1% | 77.7% |
|  | Oases | 17.2% | 78.7% | 28.1% | 72.5% |
| 150 | Trinity | 25.9% | 82.2% | 35.4% | 83.9% |
|  | Oases | 20.6% | 76.7% | 31.4% | 81.5% |
| 175 | Trinity | 27.0% | 82.1% | 35.5% | 83.2% |
|  | Oases | 21.3% | 76.1% | 31.5% | 81.2% |
| 200 | Trinity | 27.2% | 81.8% | 35.6% | 83.3% |
|  | Oases | 21.9% | 75.4% | 31.9% | 79.9% |
