# Supplementary material for: The Impacts of Read Length and Transcriptome Complexity for De Novo Assembly: A Simulation Study
Source: PLoS One. 2014 Apr 15;9(4):e94825. doi: 10.1371/journal.pone.0094825 (PMC3988101; doi:10.1371/journal.pone.0094825)
Supplement: Table S4 — Comparison of de novo assemblies on five human datasets with different numbers of spliced isoforms. (DOCX) [file pone.0094825.s004.docx]

**Table S4.** Comparison of de novo assemblies on five human datasets with different numbers of spliced isoforms based on four different measures, including the percentage of full-length reconstructed reference transcripts, false positive rate, nucleotide sensitivity, and nucleotide specificity.

| # Isoforms  Per Gene | *Do novo* Assembler | Full-length Percentage | False Positive Rate | Nucleotide Sensitivity | Nucleotide Specificity |
| --- | --- | --- | --- | --- | --- |
| 1-2 | Trinity | 22.2% | 78.9% | 58.8% | 84.4% |
|  | Oases | 15.5% | 78.6% | 36.1% | 57.3% |
| 3-4 | Trinity | 18.5% | 84.1% | 50.0% | 84.2% |
|  | Oases | 10.6% | 82.9% | 27.6% | 53.7% |
| 5-6 | Trinity | 13.5% | 90.2% | 44.9% | 83.9% |
|  | Oases | 7.0% | 86.2% | 24.9% | 49.6% |
| 7-8 | Trinity | 10.6% | 92.7% | 43.3% | 83.5% |
|  | Oases | 5.3% | 90.2% | 23.4% | 47.4% |
| 9-10 | Trinity | 8.6% | 94.4% | 41.4% | 83.3% |
|  | Oases | 4.3% | 93.6% | 21.2% | 48.8% |
